# Synergistic Utilization of Multi-Source Industrial Solid Wastes in Cementitious Materials: A Comprehensive Review

**DOI:** 10.3390/ma19051019

**Published:** 2026-03-06

**Authors:** Yang Xue, Xiaoming Liu, Qi Liang, Kaisheng Zhang, Yaguang Wang

**Affiliations:** 1 State Key Laboratory of Advanced Metallurgy, University of Science and Technology Beijing, Beijing 100083, China; 2 School of Metallurgical and Ecological Engineering, University of Science and Technology Beijing, Beijing 100083, China; d202520041@xs.ustb.edu.cn; 3 School of Civil Engineering and Architecture, Henan University, Kaifeng 475004, China

**Keywords:** solid wastes, cementitious materials, properties, hydration mechanism

## Abstract

The synthesis of cementitious binders incorporating industrial solid waste represents a strategic pathway toward achieving large-scale resource valorization. The synergistic utilization of binary and ternary solid waste systems has emerged as a prominent research field, leveraging the complementary physical and chemical attributes of diverse waste streams. This work systematically evaluates the synergistic effects within multi-component solid waste systems and analyzes their influence on the mechanical properties and hydration kinetics of cementitious matrices. Specifically, the underlying mechanisms of alkali-mediated structural evolution and sulfate-induced microstructural reinforcement are characterized to elucidate the collaborative interactions between different waste phases. Finally, the prevailing technical constraints in the application of multi-component wastes are identified, and strategic directions for future development are proposed. This study provides a vital theoretical framework for the high-volume and cost-effective utilization of industrial by-products as sustainable building materials, contributing to energy conservation and carbon footprint reduction within the construction industry.

## 1. Introduction

**Figure 1:**
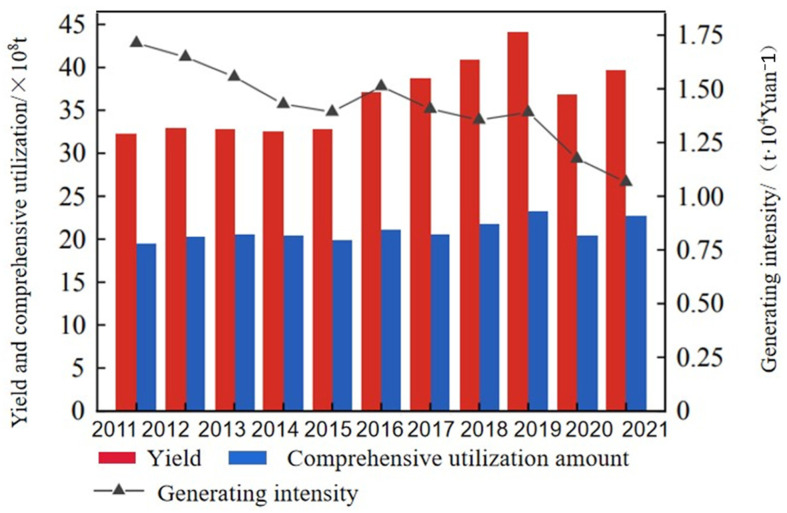
The generation, utilization, and intensity of industrial solid wastes in China from 2011 to 2021 [6].

Global industrial activities, particularly within the metallurgical, mining, and chemical sectors, discharge substantial quantities of solid waste, primarily consisting of fly ash, red mud, steel slag, and desulfurization gypsum. Currently, the annual global production of red mud reaches 170 million tons, resulting in a staggering cumulative storage of 4.2 billion tons [1,2,3]. Despite the vast accumulation of red mud, its global resource utilization rate remains below 15% [4]. Concurrently, the annual global outputs of steel slag and desulfurization gypsum have surpassed 150 million tons and 110 million tons, respectively. As industrialization accelerates, the annual generation of industrial solid waste continues to expand. Projections indicate that global solid waste production will reach a staggering 27 billion tons per year by 2050. At present, the cumulative stock of solid wastes in China is about 60 billion tons, with an annual production of nearly 3.6 billion tons and still growing at a relatively high growth rate [5]. The historical trends regarding the generation, utilization, and intensity of industrial solid waste in China from 2011 to 2021 are illustrated in Figure 1 [6]. Within the Chinese industrial landscape, the primary waste streams consist of tailings, fly ash, coal gangue, smelting waste, coal-fired slag, and desulfurization gypsum. Quantitatively, the cumulative accumulation of fly ash in China has surpassed 2.5 billion tons. Furthermore, the annual discharge of red mud has exceeded 100 million tons, resulting in a staggering cumulative storage volume of over 1.6 billion tons [7]. The accumulation of vast quantities of solid waste not only represents a significant loss of potential resources but also consumes extensive land area and imposes severe environmental burdens. Many solid wastes contain hazardous substances that can leach into and contaminate groundwater and soil resources. Granular wastes are easily blown out by wind and spread in the air. Some solid wastes also form toxic and harmful gases during transportation and disposal, which can spread to the air and affect human health [8,9,10,11]. The resource utilization of industrial solid waste has become an important cornerstone for the transformation of the global construction industry. Not only in China, according to a report by the International Energy Agency (IEA), the cement industry accounts for approximately 25% of global industrial CO_2_ emissions. Against the backdrop of global pursuit of carbon neutrality, the development of low-carbon cementitious materials based on multi-source solid waste synergistic coupling has become a common consensus in the international academic and industrial communities. This is not only to solve the common problems of land occupation and environmental pollution faced by various countries, but also a strategic requirement for achieving the greenhouse gas emission reduction targets of the Paris Agreement [12,13].

The established pathways for the resource valorization of industrial solid waste encompass a broad spectrum of applications, primarily including the synthesis of construction materials, the extraction of high-value elements, wastewater and exhaust gas remediation, and soil amendment [14]. Furthermore, these waste streams serve as sustainable precursors for the fabrication of functional catalysts [15] and high-performance adsorbents. Specifically, cementitious materials that incorporate industrial solid waste as a substitute for traditional Portland cement are characterized by high utilization efficiency, minimal technical risk, and significant cost-effectiveness. Consequently, this approach is regarded as the most viable pathway for achieving large-scale valorization of industrial by-products. Due to its superior mechanical properties, durability, and hydraulic stability, Portland cement has long served as the primary binder in the construction industry [16]. Nevertheless, its production is exceptionally carbon-intensive, with approximately 800 kg of CO_2_ emitted for every ton of cement produced. The substantial CO_2_ footprint of the cement sector positions it as a major contributor to global greenhouse gas emissions. Consequently, the development of sustainable building materials has become imperative amidst rapid economic expansion and heightening ecological concerns [17,18,19]. To address these challenges, researchers have increasingly utilized industrial solid waste as supplementary cementitious materials (SCMs) within traditional silicate-based systems. This strategy not only mitigates the high energy consumption and environmental pollution associated with conventional cement manufacturing but also facilitates the large-scale valorization of industrial by-products. However, it is important to recognize that the substitution levels achievable with a single type of solid waste remain relatively constrained [20,21,22,23].

**Figure 2:**
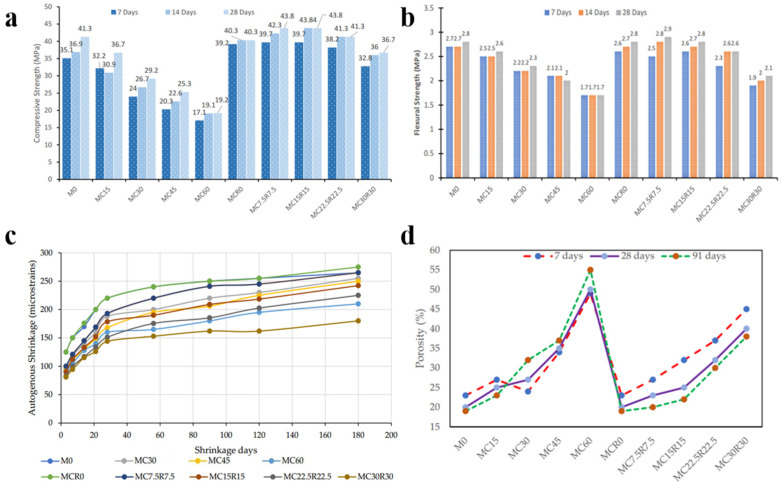
Effect of different replacement levels of GBFS with DCP and RM on (**a**) compressive strength, (**b**) flexural strength, (**c**) autogenous shrinkage, and (**d**) porosity of alkali-activated slag-based mixtures [20].

A compelling solution to these constraints lies in the synergistic coupling of diverse waste streams. For instance, Abadel et al. [20] demonstrated that the co-utilization of dehydrated cement powder (DCP) and red mud (RM) effectively optimizes material performance. As illustrated in Figure 2, while high DCP dosages typically compromise strength, the incorporation of RM, rich in active ingredients, can bolster the 28-day compressive strength by up to 42.2% (reaching 43.84 MPa) and curtail 180-day drying shrinkage by 34.5%. Furthermore, despite an initial increase in porosity due to the high-water absorption of RM, the filler effect provided by its fine particles significantly densifies the matrix during later stages of hydration. This evidence underscores multi-source waste coupling as a robust strategy for achieving a vital equilibrium between mechanical integrity and environmental sustainability.

Driven by advancing research into sustainable construction, recent studies have demonstrated that the synergistic utilization of binary or ternary solid waste systems significantly enhances the performance of cementitious materials compared to single-component systems [24,25,26]. This collaborative approach effectively mitigates the inherent drawbacks associated with individual waste types [27,28,29]. In this work, the impact of various solid wastes on the macroscopic properties of cement-based materials was systematically evaluated, and the underlying hydration mechanism and chemical interactions between multiple waste phases were analyzed in detail. In addition, existing technological limitations that hinder the large-scale application of solid waste have been identified, and strategic paths for future optimization have been proposed. This study provides an important theoretical framework for the cost-effectiveness and large-scale value-added of industrial by-products as building materials, which contributes to the energy efficiency and decarbonization of the cement industry. Finally, to overcome the limitations of single solid waste utilization, this paper proposes a preliminary collaborative evaluation framework: by analyzing the degree of overlap between “chemical activity complementarity” (such as red mud providing alkaline activated slag) and “physical stacking optimization” (such as micro fly ash filling pores), the collaborative index is qualitatively evaluated, providing a basis for screening the best combination of solid waste.

## 2. Influence of Multi-Component Solid Waste Systems on Mechanical Performance

### 2.1. Mechanical Properties of Binary Synergistic Systems

Extensive research has demonstrated that cementitious materials incorporating binary solid waste systems—composed of two waste types with complementary physicochemical properties—exhibit superior performance compared to single-waste systems. These findings suggest a significant synergistic effect between the components during the hydration process. Specifically, the aluminosilicate phases within the system are effectively activated by the presence of alkaline or sulfate-rich wastes, which accelerates the hydration kinetics and subsequently enhances the overall mechanical properties of the binder [30].

**Figure 3:**
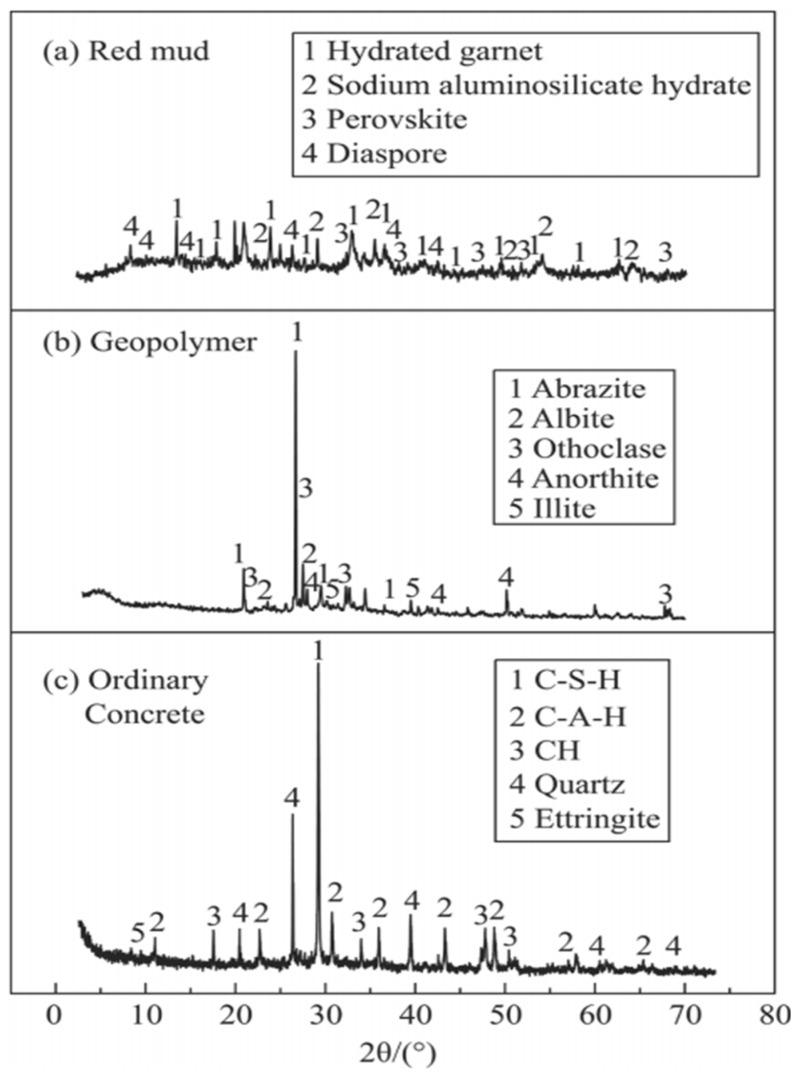
XRD patterns of raw materials: (**a**) red mud, (**b**) geopolymer, (**c**) ordinary concrete [34].

Alkaline industrial by-products, including red mud, alkali slag, and carbide slag, establish the requisite alkaline environment for the hydration of aluminosilicate-rich precursors within composite binder systems. In this environment, OH^−^ ions react with aluminosilicate components to precipitate extensive silicate-based hydration products [31]. When incorporated into multi-component systems, red mud serves a dual function [32], Beyond providing a physical filling effect that densifies the matrix, the soluble alkalis within red mud accelerate the dissolution of active silica and alumina minerals. This chemical interplay enhances the overall degree of hydration, leading to the increased formation of secondary hydration products such as AFt and calcium silicate hydrate (C-S-H) gel, which collectively reinforce the mechanical integrity of the material [33]. Liang et al. [34] synthesized geopolymer binders utilizing a red mud and granulated blast-furnace slag (GBFS) precursor system, subsequently producing geopolymer concrete as a full replacement for traditional Portland cement concrete [35]. The experimental results demonstrated that the geopolymer concrete achieved a compressive strength of 54.4 MPa, meeting the rigorous requirements for structural load-bearing components. The XRD patterns of the raw red mud and the resulting geopolymer concrete are illustrated in Figure 3. Furthermore, the composite system exhibited a refined pore structure and significantly reduced total porosity. Compared to systems incorporating red mud alone, the integration of red mud with aluminosilicate-rich precursors triggers a superior synergistic effect, which enhances the degree of alkali activation and optimizes the mechanical performance of the cementitious matrix. Zhou et al. [36] Investigated the mechanical performance of cement-based composite mortars incorporating varying proportions of steel slag and red mud. Their findings revealed that at an optimal dosage, the 28-day flexural and compressive strengths of the composite mortar increased by 11.5% and 20.6%, respectively, compared to the control group containing only 30% steel slag. Similarly, Ding et al. [37] synthesized a red mud-slag geopolymer binder with a 1:1 mass ratio. The resulting material achieved a remarkable 28-day compressive strength of 72.1 MPa, significantly surpassing the strength of binders utilizing either red mud or slag in isolation. These results underscore the potent alkaline activation effect of red mud on the reactive phases within the slag, facilitating a more robust alkali activation process.

The role of alkaline solid wastes (alkali slag and carbide slag) in the cementitious materials system is similar to that of red mud. In the early stage of hydration, the active minerals in alkali slag and carbide slag dissolve, causing an increase in the pH value of the system and providing the necessary strong alkaline environment for the system reaction [38]. Besides, the hydration reactions of silica-aluminum active substances such as silica and alumina are treated with alkaline solid wastes, including pozzolanic reactions. In the middle and later stages of hydration, alkali slag and carbide slag can also promote the dissolution of silicon aluminum substances, accelerate the rate of secondary hydration reaction, and promote the hydration of active SiO_2_ and Al_2_O_3_ to form C-(A)-S-H and needle shaped AFt [39]. The presence of AFt crystals can form a skeleton structure, and C-(A)-S-H firmly bonds AFt crystals together and fills the pores, forming a dense structure with high strength, making the structure denser and significantly improving mechanical properties [40,41]. An et al. [42] synthesized a slag-carbide slag geopolymer system. Their investigation revealed that with a 14% carbide slag addition and a water-to-binder ratio of 0.34, the geopolymer achieved a compressive strength of 31.8 MPa following a curing regimen of 32 h of steam treatment and 4 days at ambient temperature. In this system, carbide slag functions primarily as an alkaline activator, establishing the robust alkaline environment essential for alkali activation and exhibiting a potent activation effect on the slag. Nan et al. [43] evaluated the mechanical performance of binders based on steel slag and granulated GBFS across various mix proportions. Their findings confirmed a significant hydration synergy between the two slags; at the optimal ratio, both flexural and compressive strengths peaked, reaching 7.3 MPa and 20.2 MPa at 28 days, respectively. Similarly, Zhang et al. [44] developed low-carbon binders utilizing carbide slag and blast furnace slag. The results demonstrated that carbide slag significantly enhances the early-stage compressive strength of the slag mortar. Notably, the 3-day compressive strength of mortar with 2.5 wt% carbide slag was approximately 16 times higher than that of the control group, while a 5.0 wt% addition yielded a 30-fold increase. However, the rate of strength enhancement diminished at higher dosages; increasing the carbide slag content from 7.5 wt% to 10.0 wt% resulted in a strength improvement of only 19.7%, with values rising from 15.7 MPa to 18.8 MPa.

Sulfate-rich solid wastes effectively enhance the pozzolanic reactivity of aluminosilicate precursors. During the hydration process, sulfates facilitate the gradual dissolution-driven mechanisms of the amorphous glass network within these precursors, subsequently reacting with liberated calcium and aluminum ions to precipitate hydration products such as AFt [45]. As hydration progresses, the accumulation of these products—where AFt crystals interlace with hydration gels—densifies the matrix pore structure, thereby improving the overall compressive strength. Electrolytic manganese slag (EMS) serves as a representative sulfate-bearing waste that activates the latent pozzolanic potential of fly ash, steel slag, and blast furnace slag [46]. Specifically, EMS promotes the dissolution of the silicate-aluminum framework in these substances, accelerating their participation in hydration kinetics. The resulting proliferation of AFt and C-S-H gel enhances both the matrix density and mechanical performance, serving as a macroscopic manifestation of the synergistic interplay between electrolytic manganese slag and aluminosilicate-rich waste streams [47,48,49].

Extensive studies have demonstrated that the integration of binary solid waste systems into cementitious binders yields a more pronounced synergistic effect than the use of single-component wastes, leading to a substantial enhancement in compressive strength. The mechanical performance of binder systems incorporating various typical binary waste combinations is summarized in Table 1. As evidenced by the tabulated data, these composite materials exhibit robust mechanical properties, underscoring their viability for diverse engineering applications and structural scenarios. However, although Table 1 shows that most binary systems have improved strength, there are significant inconsistencies between different studies. For example, in the red clay slag system, some studies have reported significant improvements in early strength, while others have pointed out that the high-water absorption of red clay leads to a decrease in effective water cement ratio. If no additional water reducing agent is added, its early fluidity and compactness will be damaged instead. This difference is usually attributed to the non-standardization of raw material reactivity, activator concentration, and maintenance system, which limits the direct comparability between different literature conclusions.

### 2.2. Synergistic Enhancement in Ternary Solid Waste Systems

The binary solid wastes in cementitious materials were introduced in the previous chapter. However, the binary solid wastes systems of cementitious materials can usually only utilize the advantages of solid wastes characteristics, and cannot fully utilize the excellent synergistic excitation effects that may exist in multiple solid wastes. Nowadays, many scholars have found that the performance of ternary solid wastes cementitious materials systems is more excellent, and alkaline solid wastes and sulfate solid wastes may further synergistically stimulate the activity of silicon aluminum substances. The synergistic formulation of cementitious binders using ternary solid waste systems maximizes the reactive potential of diverse active components through multi-phase interactions, facilitating high-volume waste incorporation while reducing production costs [50]. This approach yields a marked improvement in mechanical performance compared to simpler systems. For instance, a potent synergy exists among converter slag, red mud, and blast furnace slag, which can elevate the strength of P·I 42.5 cement to the performance specifications of P·C 52.5 cement. Relative to binary counterparts, ternary systems develop a more densified matrix characterized by reduced porosity—a structural refinement that significantly fosters the progressive evolution of compressive strength [51]. Furthermore, ternary combinations such as gypsum-red mud-fly ash and carbide slag-fly ash-slag demonstrate superior synergistic efficiency. In these systems, red mud and carbide slag function as primary alkaline activators, accelerating the hydration kinetics of blast furnace slag and fly ash, thereby facilitating more rapid and complete participation in the hydration process [52,53,54,55].

While current research on ternary solid waste binders predominantly emphasizes mechanical performance, critical microstructural parameters—including setting time and pore distribution—are equally vital for evaluating material functionality [16,56,57]. Ternary systems typically achieve a superior degree of hydration compared to binary or single-component binders, resulting in a more densified and homogeneous network of hydration products [58,59]. Hu et al. [60] investigated the synergistic interplay within a ternary system comprising fly ash, gypsum, and steel slag. The analytical results demonstrate that the incorporation of gypsum effectively reduces the proportion of crystalline phases within the steel slag while simultaneously increasing its amorphous glass content. Similarly, the addition of fly ash promotes glass phase formation, whereas the combined integration of fly ash and gypsum significantly enhances the reactivity of the modified steel slag. These findings underscore a potent synergistic effect triggered by the mutual excitation of the three waste components.

**Figure 4:**
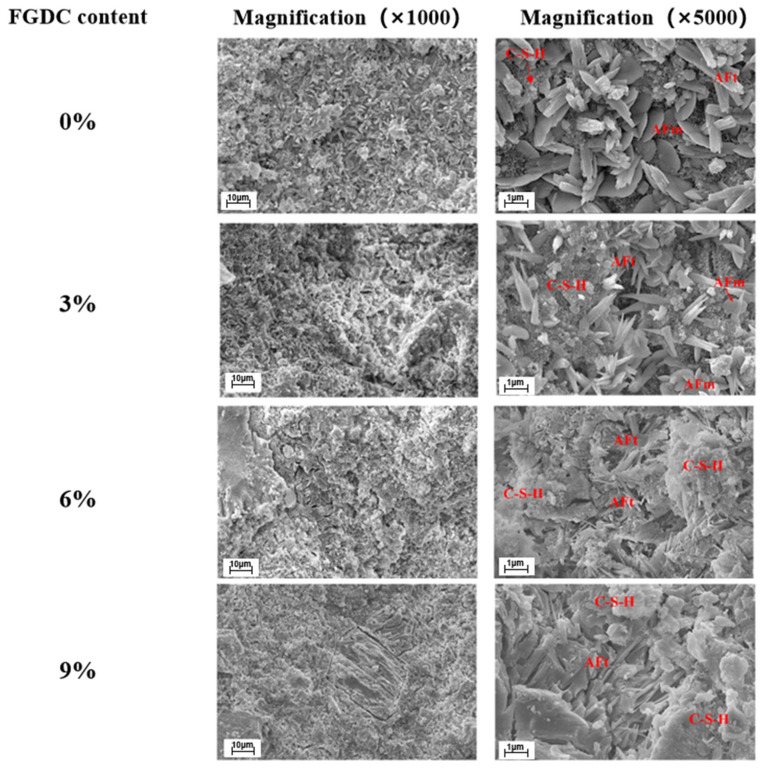
SEM images of CSAC-GGBFS mixtures with different FGDG content hydrated for 120 d [61].

Relative to single and binary systems, the ternary solid waste binder exhibits a significantly more indistinct particle morphology, characterized by an increased accumulation of C-S-H gel surrounding the precursors and the interlaced growth of AFt crystals, both of which signify an advanced degree of hydration [61]. As shown in Figure 4, the microstructure of CSAC-GGBFS mixtures with different FGDG contents after 120 days of hydration. At low magnification (×1000), the density of the hardened cement paste improved as the FGDG content increased from 0% to 6%. Specifically, gypsum-rich and porous regions within the matrix serve as preferential nucleation sites for AFt; as these porous areas typically represent structural vulnerabilities, the precipitation of AFt plays a critical role in infilling these voids and augmenting early-stage strength [62]. Furthermore, ternary waste combinations exert a substantial influence on the setting characteristics of the binders. In the gypsum-red mud-blast furnace slag system, various gypsum types can effectively accelerate the setting process. However, a retarding effect is observed when the gypsum dosage exceeds a specific threshold [63]. Chemically, Ca^2+^ from gypsum interacts with silicates, while SO_4_^2-^ facilitates the leaching of Al^3+^ and Si^4+^ ions from the precursors. This synergistic action promotes the proliferation of C-S-H gel, thereby accelerating the initial setting and hardening of the cementitious slurry [64].

The engineering applicability of the ternary systems summarized in Table 2 is determined by their distinct mechanical profiles. For instance, the alkali-activated RM-BFS-GBFS system (Sr. No. 2) and the DG-GBFS-BFS system (Sr. No. 10), which achieve 28-day compressive strengths of 56.3 MPa and 65.2 MPa respectively, are well-suited for high-performance concrete and structural components in civil infrastructure. Conversely, systems exhibiting moderate strength development, such as those incorporating electrolytic manganese residue (Sr. No. 1), are more appropriately positioned for road water stabilizer materials or as binders in cemented paste backfill for underground mining where high-volume waste incorporation is prioritized over peak strength. These application-oriented correlations transform the experimental data into a utilitarian roadmap for industrial waste valorization.

## 3. Synergistic Hydration Mechanisms Within Composite Binder Systems

### 3.1. Alkali-Mediated Structural Dissolution and Reorganization

**Figure 5:**
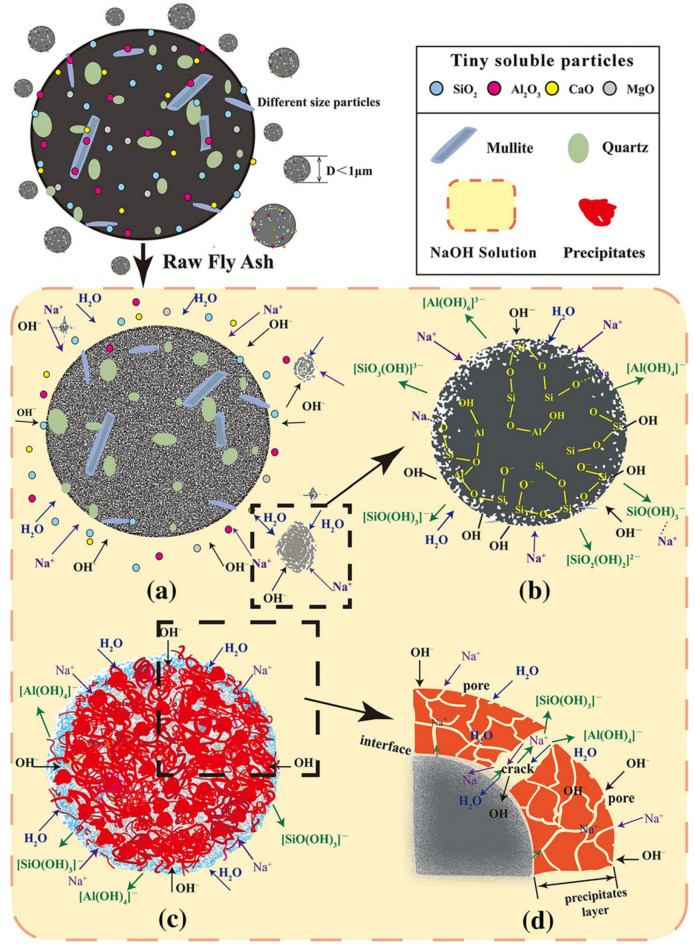
The activation mechanism of low calcium fly ash in NaOH solution: (**a**) dissolution stage, (**b**) depolymerization stage, (**c**) polycondensation and polymer gel stage, and (**d**) diffusion stage [65].

Alkaline activators play a critical role in unlocking the latent pozzolanic activity of aluminosilicate-rich solid wastes. In a highly alkaline environment, the crystalline and glassy structures of these materials undergo dissolution and structural disintegration, significantly increasing their chemical reactivity. During the synthesis of alkali-activated binders, the aluminosilicate precursors undergo a sequence of chemical transformations-including monomeric dissolution, structural reconfiguration, and subsequent polycondensation-driven by the chemical environment provided by the alkaline activator. These processes culminate in the development of a resilient, three-dimensional polymeric network. For example, under the action of OH^−^, the surface structure of fly ash is destroyed, releasing a large number of active substances and stimulating activity. The activation mechanism of low calcium fly ash in NaOH solution is shown in Figure 5 [65]. Upon contact with an alkaline solution, the soluble phases on the fly ash surface dissolve, initiating the release of reactive ionic species. Driven by the catalytic action of OH^−^, the Si-O-Si, Si-O-Al, and Al-O-Al within the fly ash glass network undergo hydrolytic cleavage. This process disrupts the stable tetrahedral frameworks of [AlO_4_]^2−^ and [SiO_4_]^−^, triggering dissolution-driven mechanisms and the subsequent formation of monomeric species such as Al(OH)_4_^−^ or Al(OH)_6_^3−^, Si(OH)^3−^ or SiO_2_(OH)_2_^2−^. These monomers undergo polycondensation via hydroxyl group interactions to form intermediate complexes, which evolve into an oligomeric sol through dehydration and condensation. Subsequently, these sol particles are interconnected by metal cations, culminating in the development of a resilient three-dimensional hydration network. As the reaction progresses, the formation of an initial hydration layer on the fly ash surface creates a diffusion barrier, temporarily hindering direct contact with the alkaline solution. Nevertheless, Na^+^ and OH^−^ ions continue to infiltrate the fly ash through micro-cracks and pores within the hydration products, allowing for sustained reaction and the outward diffusion of newly generated ionic monomers to continue the gelation process [66].

The activation effect of alkaline activators on the activity of silicon-aluminum character solid wastes is influenced by various factors. The activation effect of alkaline activators is directly proportional to the concentration of OH^−^, and is also related to the curing temperature and curing time [67]. The hydration kinetics of cementitious materials are significantly temperature-dependent; while basic hydration proceeds at ambient temperatures, elevated temperatures (25–90 °C) can further accelerate the dissolution of precursors and the subsequent precipitation of hydration products, leading to higher early-stage strength. On the other hand, for the types of alkaline activators [68], when the concentration, curing temperature, and time are the same, the activation effect of NaOH is better than that of KOH, while the activation effect of directly adding quicklime powder is better than that of Ca(OH)_2_. Wen et al. [69] found that the activation of fly ash is mainly divided into two stages. The first stage is the erosion of fly ash and the formation of alkaline silica solution or sol. During the erosion dissolution process, exposed Si atoms adsorb excess OH^−^ to form unstable structures, which leads to the loosening and release of Si-O-Si bonds [H_3_SiO_4_]^−^ and [H_2_SiO_4_]^2−^. The SiO_2_ monomers that appear during the dissolution process are re aggregated into SiO_2_ sol particles with a large number of negative charges under the action of OH^−^. The second stage is calcium and various metal cations. SiO_2_ sol particles are condensed to form hydrated silicate gel through electrostatic bond or coordination bond, which is gathered on the surface of fly ash particles. Fraay et al. [70] found that in the presence of lime solution, the minimum pH at which Si-O and Al-O bonds on the surface of fly ash particles can be broken and dissolved to produce silicon aluminum ions is 13.3. However, the pH of saturated Ca(OH)_2_ solution at room temperature is 12.5, so the activation effect of Ca(OH)_2_ solution on fly ash is not ideal at room temperature. Weng [71] made up for this deficiency by using steam pressure curing. Under the dual effects of chemical activation and high-temperature activation, fly ash exhibits high reaction activity. Qiao [72] found that excessive Ca(OH)_2_ can cause cement hydration products to precipitate early from the solution and cover fly ash particles, hindering the continuation of hydration reactions. Within a certain dosage range, when quicklime is directly added, the low degree of [SiO_4_]^−^ and [AlO_6_]^3−^ absorb OH^−^, causing the fly ash particles to carry negative charges, while the digested CaO particles carry positive charges. The two attract each other, promoting the formation of hydrated calcium silicate, hydrated calcium aluminate, and calcium carbide. Moreover, CaO reacts with water to release a large amount of heat, which can increase the system temperature and promote the activation of fly ash activity. However, it should be noted that the expansion risk associated with CaO is not solely dependent on its dosage. The reactivity, particle size distribution, and calcination history of the quicklime play critical roles in governing expansion behavior. While highly reactive or finely ground CaO can integrate into the microstructure through rapid hydration, low-reactivity or coarse particles may undergo delayed hydration, generating localized expansive stresses and subsequent microcracking in the hardened matrix [73]. Although lime can stimulate the activity of fly ash, lime is a weakly alkaline activator that is slightly soluble in water and has limited stimulation on the activity of fly ash.

In addition, silicates can be classified as alkaline activators, mainly because when Na_2_SiO_3_ is used as an activator for fly ash activity, Na_2_SiO_2_ hydrolyzes to generate NaOH, making the slurry alkaline. The pH value of the system can reach 13.1, which is higher than the pH value of saturated Ca(OH)_2_ solution at room temperature. Therefore, the essence of Na_2_SiO_3_ activated fly ash is alkaline activation. The hydrolysis equation is as follows:

SiO_3_^2−^ + 2H_2_O → H_2_SiO_3_↓ + 2OH (1)

**Figure 6:**
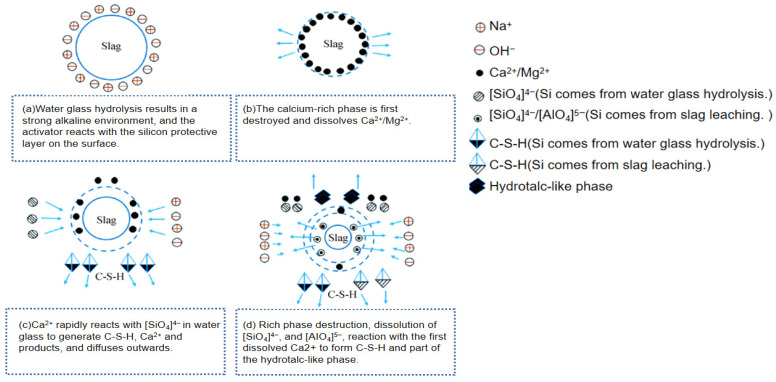
Mechanism of Sodium Silicate Water Glass Stimulating Slag [74].

The hydrolyzed silicic acid produced is gelatinous, and it is insoluble in water. Gel can react with Ca^2+^ to generate hydrated calcium silicate gel, which promotes the hydration reaction of fly ash. The activation of Na_2_SiO_3_ on the activity of fly ash is a dual activation, and the activation effect of fly ash is better than NaOH and Ca(OH)_2_. Moreover, under conditions of steam pressure curing and high temperature and pressure, the larger the dosage, the more obvious the activation effect [73]. The mechanism of sodium silicate water glass stimulating slag is shown in Figure 6 [74].

Composite alkaline activators can promote the hydration of cement and stimulate the dissociation of fly ash structure, accelerating its cracking and hydration under the combined action of sodium, calcium, and sulfur. At the same time, they provide the necessary conditions for the formation of cementitious materials, such as Ca^2+^, which can quickly improve early strength [75]. At the initial stage of the reaction, under the erosion and destruction of high concentration OH^−^, the active silicon aluminum oxide in the fly ash is continuously dissolved into oligomers such as silicon oxygen tetrahedron and aluminum oxide tetrahedron. Then Na^+^ in the system starts to combine with silicon aluminum, and polymerization reaction occurs between oligomers to continuously generate space network structure of silicon aluminate N-A-S-H gel. The newly formed sodium aluminosilicate hydrate (N-A-S-H) gel undergoes continuous polymerization, with its cumulative volume expanding as the reaction progresses. The proliferation of N-A-S-H gel serves as the primary structural backbone, significantly enhancing the compressive strength of the matrix [76]. The activation efficiency of fly ash is highly sensitive to the dosage of the composite activator [77]. At a lower dosage of 10%, the dissolution of reactive silica and alumina is restricted by the insufficient alkalinity and limited silicon sources available for the alkali activation process. As the activator concentration increases, the elevated alkalinity facilitates a more thorough dissolution of the aluminosilicate phases, promoting the development of a more intricate three-dimensional network and improving mechanical performance. However, a decline in strength is observed when the activator dosage reaches 30%. This phenomenon is likely attributed to the excessive concentration of sodium silicate, which induces an ultra-rapid reaction rate. The accelerated precipitation of hydration products creates a dense gel barrier that encapsulates the unreacted fly ash particles, thereby hindering the inward diffusion of OH^−^ and the subsequent dissolution of internal reactive components.

Alkaline activators such as NaOH and CaO were replaced by alkaline solid wastes such as red mud and calcium carbide slag. This way can not only reduce the cost of alkaline activator, but also is an effective way to use industrial wastes resources [78,79]. The incorporation of an optimal red mud dosage facilitates the dissolution of aluminosilicate phases within the cementitious system, thereby augmenting the hydration activity of the binder matrix. This process promotes the accelerated precipitation of hydration gels and AFt, which effectively infills internal micro-voids. Consequently, the resulting microstructural densification leads to a significant enhancement of both the internal structural integrity and the overall mechanical properties of the matrix [80]. The alkalinity released from activators such as red mud and carbide slag establishes the requisite high-pH environment for the hydrolytic dissolution of aluminosilicate frameworks within solid waste precursors. This process yields various monomeric species, including [H_3_SiO_4_]^−^, [H_3_AlO_4_]^2−^, and [Al(OH)_6_]^3−^, as shown in Equations (2)–(4). At ambient temperature, these metastable oligomers undergo polycondensation and diffuse from the precursor surfaces into the interstitial pores, culminating in the formation of three-dimensional C-(A)-S-H and N-A-S-H gel networks adhering to the raw material surfaces. As curing progresses, the connectivity of the gel framework is progressively reinforced, as described by Equations (5) and (6) [81]. Notably, increasing the proportion of alkaline waste elevates the OH^−^ concentration, thereby intensifying the activation of the aluminosilicate phases and accelerating the precipitation of hydration products to facilitate early-stage strength development. Nevertheless, exceeding the threshold dosage can result in an excessively high OH^−^ concentration; this triggers an ultra-rapid surface reaction that forms a dense, impermeable protective film over the unreacted precursors, effectively shielding them and inhibiting further hydration.

SiO_2_ + OH^−^ + H_2_O → [H_3_SiO_4_]^−^ (2)

AlO_2_^−^ + OH^−^ + H_2_O → [H_3_AlO_4_]^2−^ (3)

AlO_2_ + OH^−^ + H_2_O → [Al(OH)_6_]^3−^ (4)

Ca^2+^ + [H_3_SiO_4_]^−^ + [H_3_AlO_4_]^2−^ → C-(A)-S-H (5)

Na^+^ + [H_3_SiO_4_]^−^ + [H_3_AlO_4_]^2−^ → N-A-S-H (6)

At present, research on the synergistic mechanism mainly focuses on the qualitative description of hydration products, lacking in-depth analysis of the evolution process of the interfacial transition zone (ITZ) under multi-source solid waste coupling. In addition, most of the cited studies focus on 28-day compressive strength, neglecting the long-term evolution of multi-component systems in complex service environments such as carbonation resistance and chloride ion penetration resistance, which is a theoretical limitation that must be overcome before future industrial applications.

### 3.2. Sulfate-Induced Precipitation and Microstructural Reinforcement

The activation of sulfate on silicon and aluminum substances in solid wastes is mainly due to the action of Ca^2+^. SO_4_^2−^ reacts with gel products and AlO_2_^−^ to generate AFt [82,83,84]. At the same time, in alkaline environment, Al-O bond and Si-O bond break to release [AlO_4_]^5−^, [SiO_4_]^4−^. Some [SiO_4_]^4−^ in gel products is replaced by SO_4_^2−^, and the replaced [SiO_4_]^4−^ and other Ca^2+^ generate gel products, so that the activation of silicoaluminal minerals continues. The mechanism of action of sulfate activator is to ionize SO_4_^2−^, which can cause the Si-O and Al-O bonds at the reaction activation point to break and react with the active Al_2_O_3_ dissolved in the liquid phase under the action of Ca^2+^ to form AFt [85]. The chemical reaction formula is as follows:

Al_2_O_3_ + Ca^2+^ + SO_4_^2−^ + OH^−^ → 3CaO·Al_2_O_3_·3CaSO_4_·32H_2_O (7)

Partial hydrated calcium aluminate can also react with gypsum to form AFt, with the following chemical reaction formula:

3CaO·Al_2_O_3_·6H_2_O + 3(CaSO_4_·2H_2_O) + 20H_2_O → 3CaO·Al_2_O_3_·3CaSO_4_·32H_2_O (8)

Solely utilizing sulfates to activate low-calcium fly ash often results in a prolonged induction period where the system fails to solidify within 28 days, necessitating the addition of lime to supplement the requisite calcium. Conversely, in high-calcium fly ash systems, the sulfate activation mechanism predominantly involves the substitution of SO_4_^2−^ for a portion of the [SiO_4_]^4−^ units within the C-S-H gel framework. The liberated [SiO_4_]^4−^ ions subsequently react with Ca^2+^ in the external solution to precipitate additional C-S-H gel, thereby accelerating the hydration process, while the presence of free silicates further facilitates the dissolution of reactive Al_2_O_3_. Simultaneously, [SiO_4_]^4−^ species can adsorb onto the active Al^3+^ network intermediate sites on the surface of the fly ash glass, triggering the hydrolytic cleavage of Al-O and Si-O bonds [86,87,88]. Furthermore, the partial incorporation and adsorption of SO_4_^2−^ within the C-S-H structure alter its water permeability, further catalyzing the formation of the hydration products. Beyond chemical activation, the crystallization of CaSO_4_ and related calcium silicate phases induces a controlled expansion effect. This expansion effectively infills interstitial voids within the matrix, enhancing the compactness of the hardened slurry and compensating for chemical shrinkage [89].

The most commonly used sulfate activators are CaSO_4_ and Na_2_SO_4_, with the latter having a better excitation effect than the former. On the one hand, Na_2_SO_4_ has a higher solubility and higher dispersibility when reacting with Ca(OH)_2_ in the system to generate CaSO_4_. On the other hand, the reaction between Na_2_SO_4_ and Ca(OH)_2_ generates CaSO_4_ and NaOH, which enhances the alkalinity of the system. Therefore, the activation of fly ash by Na_2_SO_4_ is categorized as a dual-activation mechanism [90,91,92,93,94,95]. Wang et al. [96] posited that Na_2_SO_4_ promotes the formation of more structurally integrated fibrous and reticular hydration products compared to CaSO_4_ activation. This process results in a less dense encapsulation layer, thereby facilitating the outward diffusion of Ca^2+^ and accelerating the reaction kinetics. In high-calcium fly ash systems, the chemical interaction between Na_2_SO_4_ and Ca(OH)_2_ is further intensified, yielding additional reactive species. Furthermore, Poon et al. [97] identified a distinct correlation between the efficiency of sulfate activators and the intrinsic calcium content of the fly ash; specifically, Na_2_SO_4_ exerts a more potent activation effect on high-calcium variants, particularly during the later stages of hydration. Curing temperature also serves as a critical determinant of activation efficiency. At a constant dosage, the degree of activation is positively correlated with the curing temperature, as elevated temperatures provide the thermal energy necessary to overcome the activation barriers of the pozzolanic reactions.

The dosage of sulfate activator cannot exceed a certain range, about 0–5% [98,99,100]. Excessive Na_2_SO_4_ dosage can cause frost phenomenon, and AFt will be generated in the later stage due to the slow dissolution of active Al_2_O_3_ in fly ash, which has swelling properties [101,102]. If the amount generated is too high, microcracks will be generated inside the material, causing a decrease in strength in the later stage [103]. In recent years, the most studied systems were gypsum activated fly ash or blast furnace slag, both of which have achieved certain results. However, their addition amount is relatively low, and when the addition amount is too high, the material properties significantly decrease [104,105,106,107,108].

Sulfate based solid wastes such as desulfurization gypsum or electrolytic manganese slag can be used to activate silicon-aluminum character solid wastes. Under the action of sulfate in desulfurization gypsum, the dissolution rate of inert silicon aluminum substances in silicon-aluminum character solid wastes is increased, thereby increasing the degree of hydration reaction [109]. Ca^2+^ ions and SO_4_^2−^ ions dissolved from CaSO_4_ will further react with octahedral [Al(OH)_6_]^3−^ ions to form rod-shaped AFt. The enrichment of Ca^2+^ ions facilitate the precipitation of C-A-S-H gel and AFt, which in turn accelerates the dissolution of aluminosilicate frameworks by continuously consuming reactive SiO_2_ and AlO_2_^−^ species. Incorporating sulfate-bearing wastes, such as electrolytic manganese slag and desulfurization gypsum, typically maintains the fundamental assembly of hydration products, primarily characterized by flocculent C-S-H gel [110]. Simultaneously, these sulfate sources react with active aluminates to generate AFt, which effectively infills micropores and microcracks, thereby fostering a more densified matrix structure [111]. Furthermore, the presence of SO_4_^2−^ exerts a secondary promotional effect on activation, particularly within low-alkalinity systems [112], by catalyzing the polycondensation process and enhancing the structural integrity of the resulting polymer network. However, precise dosage control of desulfurization gypsum is critical. Specifically, the gypsum content should be restricted to below 6% [80,113] to avoid an excessive volume expansion effect that may render the matrix porous and friable, as the overproduction of AFt crystals beyond the matrix’s expansion threshold can induce internal micro-cracking. In high-calcium systems, excessive sulfate concentrations may also impede the formation of geopolymer gels, ultimately compromising the terminal mechanical strength. In addition, it is necessary to be highly vigilant about the risk of delayed ettringite formation (DEF) in sulfate activated systems. Although the initially generated AFt has the function of filling pores, if there are excessive sulfate ions remaining in the system, these ions may slowly react with the aluminate phase in the hardened matrix in the later stage of material service, leading to the expansion of secondary ettringite crystals in the confined space. The internal stress generated by delayed expansion often exceeds the tensile strength of the matrix, leading to microcracks and even powdering and peeling of the structure.

### 3.3. Interaction and Coupling Effects of Alkali and Sulfate Activators

The composite activation mechanism of alkali and sulfate on silicon and aluminum substances in solid wastes is relatively complex. In the system of alkaline and sulfate type solid wastes cementitious materials, alkaline solid wastes and sulfate type solid wastes provide OH^−^ and SO_4_^2−^, respectively. When SO_4_^2−^ and OH^−^ act together, Al-O and Si-O chemical bonds are destroyed, forming structures dominated by [SiO_4_]^4−^ and [AlO_4_]^5−^ [114]. The aluminum dissolved from the silicon-aluminum character solid wastes reacts with OH^−^ and SO_4_^2−^ to produce AFt. The formation of AFt reduces the concentration of Al in the solution, promotes the further dissolution of silicon-aluminum character solid wastes [115], and speeds up the formation rate of hydration product gel. The formation can effectively stimulate Al_2_O_3_ in the system, make the matrix more compact, and increase the hydration product content and hydration degree of the whole cementitious materials [116]. In addition, SO_4_^2−^ ions interact with the Ca^2+^ within the C-S-H or C(N)-A-S-H gel lattices, leading to the displacement of SO_4_^2−^ units. These liberated SO_4_^2−^ tetrahedra subsequently react with available Ca^2+^ to precipitate additional C-S-H or C(N)-A-S-H phases, thereby sustaining the dissolution of silicate species within the system. Concurrently, the presence of liberated SO_4_^2−^ significantly enhances the solubility of reactive aluminum-bearing phases, further accelerating the dissolution of aluminum. The resulting synergistic interplay between alkalis and sulfates optimizes the dissolution of aluminosilicates and accelerates the precipitation kinetics of hydration gels. This collaborative effect facilitates continuous hydration and promotes the formation of calcium aluminosilicate hydrates and AFt, ultimately reinforcing the mechanical integrity of the binder system [117]. However, the investigation of the reaction sequence of alkali and sulfate synergy has not been clarified, including the activation sequence, rate and formation process of various products of alkali and sulfate on silicon-aluminum character solid wastes.

## 4. Conclusions

The synergistic utilization of multi-component industrial solid wastes as supplementary cementitious materials represents a transformative approach to achieving high-volume resource valorization and carbon neutrality in the construction sector. This review demonstrates that the “synergistic effect” in binary and ternary systems is not merely a linear addition of properties but a complex interplay of chemical activation and physical optimization. The core of this synergy lies in the balanced reaction kinetics between alkaline waste activators (such as red mud) and aluminosilicate precursors (such as slag or fly ash), which facilitates the simultaneous precipitation of C-(A)-S-H and N-A-S-H gels to form a highly dense and interpenetrating microstructural network.

Specific findings indicate that the deliberate coupling of dehydrated cement powder (DCP) and red mud (RM) can satisfy both mechanical and sustainability requirements; for instance, a 7.5% DCP and 7.5% RM substitution level can bolster 28-day compressive strength by 42.2% while significantly curtailing 180-day drying shrinkage by 34.5% compared to single-waste systems. While sulfate-induced activation promotes early strength through the formation of a structural framework of ettringite (AFt), rigorous dosage control and environmental monitoring are essential to mitigate the long-term risks of delayed ettringite formation (DEF) and associated expansion-induced microcracking.

Furthermore, the integration of diverse solid wastes provides a robust pathway for the immobilization of hazardous ions through physical encapsulation within the dense gel matrix and chemical fixation via lattice substitution or ion exchange within zeolite-like N-A-S-H structures. Despite these advancements, the field currently faces limitations regarding the lack of standardized evaluation frameworks and long-term durability data under complex service environments. Future research must shift from empirical, experiment-driven optimization toward the development of predictive theoretical models based on precursor mineralogy to establish universal standards for multi-source cementitious materials. Ultimately, the strategic integration of industrial by-products into high-performance binders offers a vital theoretical and practical framework for energy efficiency and deep decarbonization within the global building industry.

## 5. Prospects

The integrated application of diverse solid waste streams offers a potent strategy to expand recycling pathways and elevate utilization efficiency, thereby fostering regional industrial symbiosis and yielding substantial economic and environmental dividends. This synergistic approach is widely recognized as a primary trajectory for the future of industrial waste valorization. Nevertheless, advancing the co-utilization of multiple solid wastes necessitates further investigative efforts in the following three key areas:

The hydration reaction process and the order of synergistic reactions of different types of multiple solid wastes in cementitious materials have not been clarified. There is no unified definition of the impact of alkaline solid wastes, silicon-aluminum character solid wastes, and sulfate solid wastes on the properties of cementitious materials. In the future, systematic research should be conducted to summarize the rules and identify the means of regulating the hydration reaction process of solid wastes cementitious materials.

Current research on multiple solid wastes cementitious materials predominantly focus on mechanical strength; however, their durability performance in aggressive environments remains a critical gap. Specifically, parameters such as carbonation resistance, chloride ingress, and freeze–thaw performance must be systematically evaluated before large-scale engineering adoption. For instance, while the refined pore structure in ternary systems typically improves permeability resistance, the high alkali content in certain wastes may influence carbonation kinetics or trigger alkali-aggregate reactions (AAR). Future efforts should prioritize establishing a long-term durability database to validate the structural reliability of these eco-friendly binders under diverse service conditions.

Typical solid wastes mostly contain harmful ions, such as Na^+^ in red mud and Mn^2+^ in electrolytic manganese slag. The prerequisite for its resource utilization is the solidification of harmful ions. Harmful ions are efficiently solidified by multiple solid wastes cementitious materials. However, the solidification mechanism of cementitious materials against harmful ions has not yet been explored. In the future, it is necessary to conduct relevant research on the migration, transformation, and solidification processes of harmful ions in cementitious materials.

## Figures and Tables

**Table 1:** The compressive strength of cementitious materials with typical binary solid wastes.

Sr. No	RM	SS	GBFS	FA	Others	Materials	CS (MPa)	Ref.
1	√		√			CM	16.7 (28 d)	[31]
2	√				PG	Mortar	15.2 (28 d)	[32]
3	√		√			GC	54.4 (28 d)	[33]
5				√	MM	CM	55.1 (28 d)	[34]
6	√	√				Mortar	39.8 (28 d)	[36]
7	√	√				CM	37.2 (28 d)	[37]
9		√			MM	CM	53.1 (28 d)	[39]
11		√			SF	CM	43.6 (28 d)	[40]
12		√			PG	CM	29.3 (28 d)	[41]
13			√		SF	Mortar	34.1 (28 d)	[42]
14			√		AS	GCM	31.8 (28 d)	[43]
15		√	√			CM	20.2 (28 d)	[44]
16			√		PG	CM	58.2 (28 d)	[45]
17					MS and EMR	RBM	9.1 (7 d)	[46]
18				√	EMR	CM	10.1 (28 d)	[47]
19		√			EMR	CM	43.5 (28 d)	[48]

CS: Compressive Strength; RM: Red Mud; SS: Steel Slag; GBFS: Granulated Blast-Furnace Slag; FA: Fly Ash; PG: Phosphogypsum; CM: Cementing materials; GC: Geopolymer Concrete; GCM: Geopolymer Cementitious Materials; MM: Modified Metakaolin; AS: Acetylene Sludge; SF: Silica Fume; EMR: Electrolytic Manganese Residue; MS: Municipal Solid Wastes Incineration Bottom Residue; √: This solid waste is one of the raw materials.

**Table 2:** Application of different ternary solid wastes in cementing materials.

Sr. No	RM	DG	AS	FA	SS	Others	Material	CS (MPa)	Ref.
1	√		√			EMR	RBM	5.7 (7 d)	[50]
2	√				√	BFS	CM	56.3 (28 d)	[51]
3	√			√		PG	RWSM	4.7 (7 d)	[52]
4			√	√		GBFS	CM	25.9 (28 d)	[53]
6		√		√		CG	CM	34.1 (28 d)	[54]
7	√				√	GBFS	GM	12.8 (28 d)	[55]
8	√	√		√			CM	3.4 (28 d)	[56]
9	√		√			AACM	AACM	18.2 (28 d)	[16]
10		√			√	GBFS	ASWC	65.2 (28 d)	[57]
11		√	√			GBFS	ASWCM	33.2 (28 d)	[58]
12		√			√	BFS	ASWC	55.5 (28 d)	[59]
13			√	√		TG	AACM	12.1 (28 d)	[60]
14		√		√	√		ASWCM	50.4 (28 d)	[61]
15		√				GBFS and EFS	ASWCM	39.0 (28 d)	[62]
17	√					PG and GBFS	GM	9.3 (28 d)	[63]
18	√		√			MSWIFA	GCM	11.7 (28 d)	[64]

AACM: Alkali-Activated Cementing Materials; ASWC: All Solid Wastes Concrete; ASWCM: All Solid Wastes Cementitious materials; GM: Grouting Material; RWSM: Road Water Stabilizer Material; CG: Coal Gangue; TG: Titanium Gypsum; EFS: Electric Furnace Slag; DG: Desulphurization Gypsum; √: This solid waste is one of the raw materials.

## Data Availability

No new data were created or analyzed in this study. Data sharing is not applicable to this article.
